# May Ibrutinib Have Activity in Respiratory Complications by SARS-CoV-2? Clinical Experience in a Patient with Chronic Lymphocytic Leukemia

**DOI:** 10.3390/healthcare9010078

**Published:** 2021-01-15

**Authors:** Javier Molina-Cerrillo, Juan Marquet-Palomanes, Teresa Alonso-Gordoa, Javier López-Jiménez, Enrique Grande

**Affiliations:** 1 Medical Oncology Department, University Hospital Ramon y Cajal, 28034 Madrid, Spain; 2 The Ramón y Cajal Health Research Institute (IRYCIS), CIBERONC, 28034 Madrid, Spain; jmarquet88@gmail.com (J.M.-P.); jljimenez@salud.madrid.org (J.L.-J.); 3 Medical School, Alcalá University, 28805 Madrid, Spain; 4 Hematology Department, University Hospital Ramon y Cajal, 28034 Madrid, Spain; 5 MD Anderson Cancer Center, 28033 Madrid, Spain; egrande@oncomadrid.com

**Keywords:** SARS-CoV-2, COVID19, ibrutinib, BTK

## Abstract

COVID-19 is affecting many countries all around the world. Unfortunately, no treatment has already been approved for the management of patients infected by SARS-CoV-2. It seems that SARS-CoV-2 can induce the activation of an exaggerated immune response against itself according to different mechanisms that are not really well known. Inflammatory interleukins, such as IL-6 among others, play a central role in this uncontrolled immune response. There is a strong rational under ibrutinib use in in the treatment of immune-based diseases, such a as GVHD or RA. Ibrutinib achieves a reduction in the production of TNFα, IL1, IL-6 and Monocyte chemo-attractant protein-1 (MCP-1) by neutrophils and macrophages, that are key players in keeping the inflammatory process. We present our clinical experience about ibrutinib use in ARDS secondary to SARS-CoV-2 in a patient with chronic lymphocytic leukemia (CLL).

## 1. Introduction

SARS-CoV-2 is a member of the Betacoronaviruses family that is causing a global pandemic status not seen for decades. We know that angiotensin-converting enzyme 2 (ACE2) is the key pathway for the entry of the virus into the cell [1]. Infected patients present different symptoms, ranging from completely asymptomatic to the development of acute respiratory distress syndrome (ARDS) requiring admission to the ICU [2]. The reason why this infection affects some patients more aggressively than others is not clearly known. Today, it is considered that SARS-CoV-2 leads a systemic disease, and it seems that severe damage to target organs could be mediated primarily by an exaggerated and inadequate immune response. The immune response generated by the virus may enhance an increased production of TNFα, IL-1 and IL-6, among other pro-inflammatory cytokines [3]. IL-6 is mainly secreted by macrophages, fibroblasts, endothelium, and lymphocytes regulating a large part of cells of the innate and adaptive immune system. Additionally, the hematopoietic cells increase the VEGF production stimulating the angiogenesis and leading to T cells differentiating to Th17 and Treg, the activation of B cell, and fibroblast stimulation promoting stroma production [4]. Overall, this process maintains the inflammatory response and perpetuates damage to target organs including lung, liver, heart, and kidneys, among others.

Upon release into the extracellular space, IL-6 requires binding to its specific receptor, IL6R, on the effector cell. Once this binding has occurred, several intracellular signaling cascades are triggered to regulate gene expression, mainly Pi3K/AKT/mTOR, RAS/RAF/MAPK, and the JAK/STAT3 pathways [5].

The main rescue treatment strategies in seriously ill patients have focused on these proliferation pathways with the administration, as standard or under clinical trials, of monoclonal antibodies against IL-6, such as tocilizumab (NCT04317092) or even JAK/STAT tyrosine kinase inhibitors like ruxolitinib (NCT04334044).

## 2. Clinical Case

In this clinical case, we present the use of a Bruton’s tyrosine kinase inhibitor like ibrutinib in ARDS secondary to SARS-CoV-2 infection. The patient was a 72-year-old man diagnosed in 2017 with chronic lymphocytic leukemia (CLL) who was under ibrutinib therapy due to the presence of the TP53 gene mutation. The patient reached a partial response 3 months before starting ibrutinib treatment. This response is maintained at the present time. No dose reductions were required presenting only as main toxicities grade I diarrhea and grade I myalgias recovered with symptomatic treatment. As medical history, the patient presented well controlled hypertension with enalapril 20 mg per day since 2006 and dyslipidemia treated with pravastatin 10 mg per day since 2004. The patient did not present other comorbidities or concomitant therapies.

**Figure 1:**
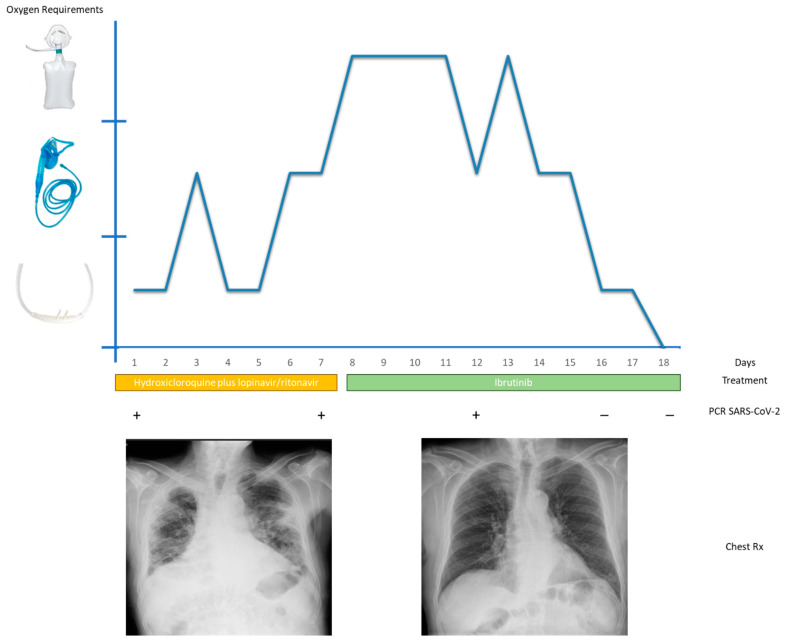
Patient’s clinical behavior according Rx evolution and oxygen requirements.

In September 2020, the patient discontinued ibrutinib by his own decision due to the coronavirus outbreak. Around ten days later, he started presenting with suspicious symptoms which consisted of cough, odynophagia and fever and was referred to the emergency department. The study was approved by the institutional ethics board of Ramon y Cajal Universitary Hospital. Code: HRC COVID 19. The patient was hemodynamical stable with an oxygen saturation of 93% when breathing ambient air. Chest X-ray showed right basal pneumonia. The blood test showed: D-Dimer 1100 ng/mL, C-reactive protein 235 mg/dL, Lactate dehydrogenase 451 U/L, lymphocytes 5100/mm^3^ with no other alterations in blood count. PCR assay confirmed the clinically suspected infection of SARS-CoV-2. Finally, the patient was admitted to the hospital and treatment with hydroxicloroquine 200 mg every 12 h and lopinavir/ritonavir 400/100 mg every 12 h was initiated. At that time, ibrutinib was still stopped. The patient worsened clinically with hypoxic respiratory failure. On the 8th day after the onset of symptoms, the patient required high-flow oxygen therapy and radiological findings were compatible with bilateral interstitial pneumonia. Blood tests showed a remarkably increase of inflammatory parameters with a serum ferritin of 751 ng/mL, C-reactive protein of 311 mg/dL, and IL-6 levels of 40 pg/mL in plasma. Other IL such as IL 8 was 24 pg/mL and TNFα of 7.1 pg/mL. At that time, and given the difficulty of managing the ARDS, the medical team decided to interrupt hydroxicloroquine and lopinavir/ritonavir and reintroduce ibrutinib at the standard dose of 420 mg per day. Three days later, the patient improved significantly along with the oxygen requirements. Seven days later, the chest X-ray showed a significant improvement of the pneumonia and inflammatory serum markers decreased dramatically to C-reactive protein 51 mg/dL, IL6 0.1 pg/mL and ferritin 210 ng/mL. Other IL did not suffer significant changes (Figure 1). Eighteen days after his admission, with two PCR assays separated 48h that did not detect SARS CoV-2, the patient was discharged. Currently, the patient remains completely asymptomatic and continues with ibrutinib treatment.

## 3. Discussion

Bruton tyrosine kinase is a member of TEC Kinases. Differently to what it might be expected, the TEC kinase family is not only expressed in the adaptive immune system, but also in the innate system. Ibrutinib is a selective inhibitor of this family of tyrosine kinases. Its use is approved for hematological malignancies, such as CLL, mantle cell lymphoma or Waldenström’s macroglobulinemia [6].

Ibrutinib has a previously established immunomodulatory role in the treatment of immune-based diseases. It has been approved for the treatment of patients with graft-versus-host disease after allogeneic stem cell transplantation, and even in some autoimmune diseases, such as rheumatoid arthritis [7,8]. Ibrutinib achieves a reduction in the production of TNFα, IL1, IL-6, and Monocyte chemo-attractant protein-1 (MCP-1) by neutrophils and macrophages, which are key players in maintaining the inflammatory process. This drug is also able to prevent the production of VEGF by endothelial cells for the inhibition of angiogenesis and prevents fibroblasts from producing extracellular matrix and metalloproteases, such as MMP-9, thereby hindering fibrosis and endothelial damage [6,9]. Furthermore, ibrutinib has the ability to inhibit the hematopoietic cell kinase (HCK), expressed in macrophages and neutrophils, that regulates the cell survival and proliferation in response to IL6 and related cytokines [10].

Moreover, BTK-mediated phosphorylation of NLRP3 plays an important role in enabling the assembly of the NLRP3 inflammasome, allowing for downstream activation and IL-1β production. Nowadays, it is unclear whether BTK plays a role on activation or inhibition of NLRP3 inflammasome activity, but this mechanism of action may be interesting to explore in patients who develop respiratory complications from COVID 19 [11].

It is also postulated that the TEC kinase family regulates the activation of cytokine receptors, such as CXCR4/CXCR7. Its inhibition is key when it comes to controlling the inflammatory response, reducing the activation, migration and adhesion of cells of the innate immune system. In addition, this axis is key to the response of effector inflammatory cells. This mechanism could also be involved in resistance to other therapies [6].

There is in vivo evidence of decreased lung damage in influenza mice treated with ibrutinib. The authors describe a significant reduction in alveolar lavage of pro-inflammatory cytokines TNFα, IL1, IL-6, MCP-1, and MMP-9 in mice treated with ibrutinib compared with controls. These mice were more likely to survive by keeping a greater lung capacity [3]. The results of this study support the rational that, on the one hand, the pro-inflammatory cytokine response causes a lung damage in viral pneumonias and, on the other hand, the ability of ibrutinib to reverse this damage.

Furthermore, in a recently published manuscript, Treon et al. report six patients with Waldenström’s macroglobulinemia treated with ibrutinib and infected by SARS-CoV-2. One of them, presented ARDS. The authors decided to increase the ibrutinib dose in these patients achieving respiratory symptomatology improving [12].

Moreover, Lin et al. published a case report regarding a patient with CLL treated with ibrutinib who develop ARDS and required mechanical ventilation. The authors continued ibrutinib and finally the improvement in patient´s clinical behavior allowed him to be extubated nine days later [13].

For this reason, ibrutinib and its immunomodulatory ability on immune cells and the secretion of inflammatory cytokines, have an important rational as a potential treatment for clinical complications associated with excessive immune activation by SARS-CoV-2, such as ARDS.

This case report and the rationale developed should be interpreted with caution due to its limitations. This case is only the communication of a patient, and we do not know the impact that other drugs received could have had on the positive final outcome of the patient. However, there are prospective trials that attempt to demonstrate the efficacy of BTK inhibitors in patients with COVID 19 and no hematological diseases (NCT04439006) or with B-Cell Malignancies (NCT04665115). Moreover, BTK inhibitors have toxicities that must be considered and assessed before starting. Among the most frequent are musculoskeletal pain, upper respiratory infection, bruising, rash, nausea, neutropenia, thrombocytopenia, or anemia. Therefore, until the results of these trials are available, only hypothesis can be generated. BTK inhibitors should not be used routinely in patients who develop ARDS. In patients under treatment with BTK inhibitors for hematological neoplasms, these drugs should be used with caution, especially in severe complications by SARS-CoV-2 infection.

## 4. Conclusions

We present the case of a patient diagnosed with CLL and ARDS secondary to COVID-19 that presented signs and symptoms relief after the reintroduction of ibrutinib. More research and even the development of clinical trials analyzing the treatment with BTK inhibitors in this setting of patients are needed.

## Data Availability

Not applicable.
